# The association of unwanted pregnancy and social support with depressive symptoms in pregnancy: evidence from rural Southwestern Ethiopia

**DOI:** 10.1186/1471-2393-13-135

**Published:** 2013-06-24

**Authors:** Yohannes Dibaba, Mesganaw Fantahun, Michelle J Hindin

**Affiliations:** 1 Department of Population & Family Health, College of Public Health and Medical Sciences, Jimma University, Jimma, Ethiopia; 2 School of Public Health, College of Health Sciences, Addis Ababa University, Addis Ababa, Ethiopia; 3 Johns Hopkins Bloomberg School of Public Health, Johns Hopkins University, Baltimore, MD, USA

**Keywords:** Depressive symptoms, Social support, Pregnancy, Violence, Food insecurity, Ethiopia

## Abstract

**Background:**

Depression in pregnancy has adverse health outcomes for mothers and children. The magnitude and risk factors of maternal depression during pregnancy is less known in developing countries. This study examines the association between pregnancy intention, social support and depressive symptoms in pregnancy in Ethiopia.

**Methods:**

Data for this study comes from a baseline survey conducted as part of a community- based cohort study that involved 627 pregnant women from a Demographic Surveillance Site (DSS) in Southwestern Ethiopia. The Edinburgh Postnatal Depression Scale (EPDS) was used to measure depressive symptoms during pregnancy. Data on depressive symptoms, pregnancy intention, social support and other explanatory variables were gathered using an interviewer-administered structured questionnaire. The association between independent variables and depressive symptom during pregnancy was assessed using multivariable logistic regression.

**Results:**

The prevalence of depressive symptoms during pregnancy was 19.9% (95% CI, 16.8-23.1), using EPDS cut off point of 13 and above. The mean score on the EPDS was 8, ranging from 0 to 25 (SD ±5.4). Women reporting that the pregnancy was unwanted were almost twice as likely to experience depressive symptoms compared with women with a wanted pregnancy. (Adjusted Odds Ratio (AOR) = 1.96, 95% Confidence Interval (CI) 1.04-3.69) Women who reported moderate (AOR = 0.27; 95% CI 0.14-0.53) and high (AOR = 0.23, 95% CI 0.11-0.47) social support during pregnancy were significantly less likely to report depressive symptoms. Women who experienced household food insecurity and intimate partner physical violence during pregnancy were also more likely to report depressive symptoms.

**Conclusion:**

About one in five pregnant women in the study area reported symptoms of depression. While unwanted pregnancy increases women’s risk of depression, increased social support plays a buffering role from depression. Thus, identifying women’s pregnancy intention and the extent of social support they receive during antenatal care visits is needed to provide appropriate counseling and improve women’s mental health during pregnancy.

## Background

Mental health problems, such as depression, rank among the top causes of disability among women worldwide; however, they still remain inconspicuous as a component of reproductive health care [1]. Several studies have shown that depression, anxiety, and stress in pregnancy are risk factors for adverse outcomes for women and children. Depression places pregnant women at greater risk for inadequate prenatal care, increased alcohol use and poorer weight gain in pregnancy [1-3]. The adverse intergenerational effects of maternal depression on children’s health, development, and behavior are also well documented [3-5]. For instance, a meta-analysis involving studies from both developed and developing countries showed that antenatal depression is associated with preterm birth, intrauterine growth restriction, and low birth weight [6]. A study from Ethiopia found that antenatal common mental disorders such as depression, anxiety and somatic symptoms are associated with prolonged labor, delayed initiation of breast feeding and more diarrheal episodes for infants [7]. Moreover, antenatal depression is a predictor of postnatal depression--women who are depressed during pregnancy have a higher risk of developing depression during the postpartum period [8,9].

We focus on depressive symptomatology in pregnancy due to its adverse consequences for mothers and children. Unlike postnatal depression, which is increasingly recognized as an important public health issue in low-income countries, antenatal depression and its effects on maternal and child health is relatively less studied. Estimates of the prevalence of antenatal depression vary substantially. A recent systematic review of studies from developed countries found that antenatal depression affects 13% of pregnant women in the second and 12% of women in third trimester [10]. Studies from South Asia and Latin America have reported rates of 20% and higher [11-14]. Evidence from developing countries suggests that depression during pregnancy is common-- in Sub-Saharan Africa prevalence ranged from 8.3% to 39% [15-18]. In Ethiopia, its magnitude and risk factors are not well known.

For most women, pregnancy is a time of positive expectation, but may also be a time for psychological and physiological challenges. It is accompanied by hormonal changes and can represent a time of increased vulnerability for the onset or return of depression [19]. However, the high prevalence of maternal depression in poor countries may be related to women’s exposure to several depression-related risk factors, including poverty [12,17,20], low social support [21-24], domestic violence [25-27], HIV/AIDS [28,29] and reproductive health outcomes and behaviors such as high parity, unwanted pregnancy, unsafe abortion, infertility, and pregnancy complications [30-33].

The intersection of unintended pregnancy and maternal mental health is not well studied in developing countries. The few studies that considered the influences of unintended pregnancy have shown that women with an unintended pregnancy are at an increased risk of depression during pregnancy than women with intended pregnancies [32-37]. Unintended pregnancy may increase a woman’s exposure to psychosocial stressors, decrease social support provided to her by the partner, increase her level of depressive symptoms, and decrease her overall life satisfaction [34-36]. Some studies show no association between unintended pregnancy and depressive symptoms during pregnancy [11,38].

Social support has the potential to play a protective role by buffering the impacts of life stress on emotional wellbeing of the woman during pregnancy [24,39,40]. Studies have shown that social support plays a buffering role from stressful life events by providing resources, support and strength during pregnancy. Understanding women’s pregnancy intention and the extent of social support they receive may help to improve healthy behaviors during pregnancy and consequently better maternal and neonatal health outcomes. This study attempts to examine the associations between pregnancy intention, social support and depressive symptoms among pregnant women in Ethiopia, where there are high levels of unintended pregnancy (estimated at 32% in 2011^a^) and women have low partner support during pregnancy [41].

## Methods

### Study setting and sample

The study took place in a Demographic Surveillance Site (DSS) in Gilgel Gibe Dam area in Southwestern Ethiopia. The Gilgel Gibe DSS is located at about 260 km southwest of Addis Ababa (the capital), and is used to collect vital events data through an update of multiple times in a year. The DSS area has a population of over 55,000, a crude birth rate of 35 per 1000, and a population growth rate of about 2.7% per annum by 2012^b^. Data collection at the site is done by Jimma University.

Data for the present study comes from a baseline survey conducted as part of a population-based cohort study in which pregnant women were identified and followed to examine factors that influence birth outcome. The outcome variable for the present analysis is maternal depression during pregnancy. All pregnant women in their 2^nd^ and 3^rd^ trimester living in the eleven kebeles^c^ (Villages) in Gilgel Gibe DSS area were targeted for participation. Six hundred twenty seven pregnant women were identified from the DSS registration and from the records of Health Extension Workers who work in each village. A baseline survey was conducted from June to July 2012 on 627 pregnant women.

A structured questionnaire was developed and administered to all study participants (study tool attached as Additional file 1). The questionnaire was first developed in English and then translated and back translated to Oromo – a local language spoken in the study area. Ten trained female interviewers with a minimum of diploma-level education collected the data. They had five days of training on how to administer the questionnaire, practice interviewing and role-plays, and how to deal with ethical issues. After the training, interviewers undertook a pilot study, and information from the pilot study was used to finalize the questionnaire. Data on depressive symptoms, pregnancy intention, social support and other explanatory variables were gathered using an interviewer-administered structured questionnaire. All study participants were interviewed at their home in private area. Ethical approval was obtained from the College of Health Sciences, Addis Ababa University. Moreover, support letters were obtained from regional, zonal and district health offices and kebele (village) administrations were informed about the study. Participants were asked for informed consent, and participation in the study was fully voluntary.

### Measurements

Depressive symptoms were measured using the Edinburgh Postnatal Depression Scale (EPDS), which describes depression as cognitive and affective features that last for at least one week, including the inability to laugh, the inability to look forward to things with enjoyment, blaming oneself unnecessarily, anxiety or worry, being scared or panicky, the inability to cope, difficulty sleeping, feeling sad or miserable, crying, and thoughts of harming oneself [42]. The EPDS is widely used and has been validated for use during pregnancy in different countries and settings [43-47], including urban and rural Ethiopia [45,48]. A psychiatrist checked the translation and back translation of the depression questions. Moreover, considering the difficulty in understanding items 1 to 3 of the depression scale (EPDS), we used examples suggested and applied by Hanlon and colleagues in their validation study in Butajira (Central Ethiopia) [45]. The items were scored on a scale of 0–3, allowing a total score ranging from 0 to 30. The internal consistency of the EPDS was tested using Cronbach alpha and was found to be 0.85. Like other previous studies that used EPDS cut of point of 13 and above [14,17], we used a cut of point of 13 and above on the scale to identify women with depressive symptoms. No other measures of mental health problems, such as anxiety and stress, were collected in this study.

The key independent variable was pregnancy intention. Women were asked to recall their feelings at the time they became pregnant: “At the time you became pregnant, did you want to become pregnant then, did you want to wait until later, or did you not want to have any (more) children at all?” The responses were categorized as (1) wanted then “wanted” (2) wanted to happen later “mistimed” and (3) did not want at all “unwanted”.

Social support was measured using the Maternity Social Support Scale (MSSS) developed by Webster and colleagues [49]. The scale contains six items and includes questions on family support, friendship network, help from spouse, conflict with spouse, feeling controlled by spouse, and feeling unloved by spouse. Each item was measured on a five-point Likert scale and a total score of 30 was possible. We classified social support in to three categories; high social support (for scores 24–30), medium social support (18–23) and low social support (below 18) categories. The internal consistency of the scale was tested using Cronbach’s alpha and was found to be 0.74.

We also considered several other explanatory variables based on previous studies including age (coded as 15–24, 25–34 and 35–49 years), women’s education (none, primary and secondary and above), occupation (housewife, farmer and employed in other services), wealth index, parity, history of miscarriage or stillbirth, perceived work burden during pregnancy, intimate partner violence, and household food security. The wealth index was computed from ownership of the following household assets: radio, television, electricity, toilet, farm land, and animals such as cattle, sheep, and goats. Principal Component Analysis (PCA) was conducted and the resulting index was divided into three categories representing poor, middle and wealthy.

Women were asked whether they have experienced a miscarriage or stillbirth in their lifetime to measure any experience of pregnancy loss. All women participating in the study were asked about the type of physical work they did in the seven days before the survey, and whether they perceived that the work was ‘difficult’, ‘moderate’ or ‘easy’ for them. Intimate partner violence was measured by asking women whether they have ever been beaten during the current pregnancy by their husbands or partners.

Household food insecurity was measured with a six- item scale based on previously validated measures in developing countries. Women were asked whether because food ran out or money was not enough to buy food, in the last 3 months, they: (1) worried about running out of food, (2) ran out of food, (3) reduced the variety of food for their children, (4) did not have enough food to give their children to eat, (5) spent the whole day without food, and (6) or anyone else in the household had ever had to ask others for food or money to buy food. For each item, ‘yes’ was coded with “1” and no coded as “0” and a summative index of food insecurity was created. Households were classified as ‘food-insecure’ if the respondent answered affirmatively to two or more of the six household food security questions. The scale had an internal consistency (Cronback’s alpha) of 0.85.

### Data analysis

Data were analyzed using STATA Version 11. First, frequency distributions of the characteristics of study population were tabulated. Next, bivariate analysis was done to compare depressive symptoms by study characteristics using Chi-square tests. Variables were entered into multivariate models based on their association in the bivariate analysis (at P < 0.20) including almost all variables that were expected to be associated from the literature review. Multivariable logistic regression was done to identify factors that are independently and significantly associated with depression during pregnancy. Odds ratios and 95% confidence intervals are reported.

## Results

Of the 627 women targeted for inclusion in the study, 622 were successfully interviewed (99% response rate). The mean age of study participants was 26 years, and ranged from 14 to 40 years (SD ±5.02). Nearly all (99%) of the respondents were married, 72% had no formal education, 77% were housewives, and 76% lived in rural areas. The median gestational age of the participants was 7 months. The average number of children ever born was 3.9, and nearly one-third (32.6%) had given birth to 5 or more children. With regards to pregnancy intention, 59% of women reported that their current pregnancy was wanted, while 28% and 13% of women their current pregnancy was mistimed and unwanted respectively. Forty-one percent of women reported food insecurity during pregnancy (Table 1).

**Table 1:** Description of study participants, Southwestern Ethiopia, 2012

**Variables**	**Number**	**Percent**
Age		
15-24	207	33.3
25-34	360	57.9
35+	55	8.8
Educational status		
No formal education	447	71.9
Primary	146	23.5
Secondary & above	29	4.7
Residence		
Rural	475	76.4
Urban	147	23.6
Marital status		
Currently married	618	99.4
Widowed or divorced	4	0.6
Occupation		
House wife	484	77.8
Farmer	92	14.8
Employed/family business	46	7.4
Trimester of pregnancy		
Second	231	37.1
Third	391	62.9
Household food security status		
Food Secure	365	58. 7
Food insecure	257	41.3
Pregnancy Intention		
Wanted	367	59.0
Mistimed	175	28.1
Unwanted	80	12.9
Parity		
0	84	13.5
1-2	163	26.2
3-4	172	27.6
5^+^	203	32.7
Total	622	100

The overall prevalence of depressive symptoms among the pregnant women was 19.9% (95% CI, 16.8-23.1). The mean score on the EPDS was 8, and ranged from 0 to 25 (SD ±5.4). Bivariate analysis showed that the prevalence of depression during pregnancy did not vary by age, wealth index, parity and trimester of pregnancy. However, the prevalence of prenatal depression varied by education, occupation, pregnancy intention, social support, perceived work burden, intimate partner physical violence, food security status and previous experience of miscarriage or stillbirth. Considering educational status, a relatively higher proportion of women with secondary and above level of education (24%) reported depression than women with no education (22%) or with primary education (11.6%). The prevalence of depressed mood in pregnancy also varied by pregnancy intention--35% of women with an unwanted pregnancy reported depressive symptoms as compared to 16% of women with wanted pregnancy. Moreover, women who scored 13 and above on the EPDS scale were more likely to have low social support, high work burden, and be farmers by occupation. Women with a high score on the EDPS were also more likely to have experienced intimate partner violence, food insecurity, and have previous history of miscarriage or stillbirth. Social support was inversely related to depressive symptoms with women reporting high social support being less likely to have depressive symptoms (Table 2).

**Table 2:** Prevalence of maternal depressive symptoms by women’s pregnancy intentions, social support and other characteristics, Southwestern Ethiopia, 2012

**Variables**	**Depressive symptoms (EPDS ≥13)**		**% with depressive symptoms**	**P**
	**No**	**Yes**		
Pregnancy Intention				
Wanted	307	60	16.4	0.001
Mistimed	139	36	20.6	
Unwanted	52	28	35.0	
Social support				
Low	30	36	53.0	0.001
Medium	253	53	22.6	
High	215	35	16.8	
Age				
15-24	171	36	17.4	0.53
25-34	284	76	21.1	
35+	43	12	21.8	
Educational status				
No formal education	347	100	34.7	0.02
Primary	129	17	11.6	
Secondary & above	22	7	24.1	
Occupation				
Housewife	411	71	14.7	0.001
Farmer	51	40	44.0	
Employed/family business	36	13	26.5	
Wealth tertile				
Poor	165	43	20.7	0.17
Middle	159	48	23.2	
Rich	174	33	15.9	
Parity				
0	70	15	17.9	0.69
1-2	134	29	17.8	
3-4	140	31	18.0	
5^+^	154	49	24.1	
Trimester of pregnancy				
2^nd^	186	45	19.5	0.83
3^rd^	312	79	20.2	
History of miscarriage/stillbirth				
No	462	103	18.2	0.001
Yes	36	21	36.8	
Presence of domestic violence				
No	484	112	18.8	0.001
Yes	14	12	46.2	
Household food security				
Food Secure	336	29	8.0	0.001
Food insecure	162	95	37.0	
Perceived work burden				
Difficult	109	43	28.3	0.01
Moderate	185	41	18.1	
Easy	204	40	16.4	
Total	498	124	19.9	

Women who did not want the current pregnancy were nearly twice as likely as women who wanted the pregnancy to experience depression during pregnancy (Odds Ratio (OR) = 1.96, 95% CI: 1.04-3.69). Women who reported a mistimed pregnancy did not differ significantly from those who wanted the pregnancy in prenatal depression. The level of social support was strongly associated with depression during pregnancy. Women with high social support were significantly less likely to experience depression during pregnancy compared with women who had high levels of social support (OR: 0.23, 95% CI 0.11-0.47). Those with moderate score on the social support scale were also less likely (OR: 0.27, 95% CI 0.14-0.53) to experience depression during pregnancy. With regards to occupation, women engaged in farming (OR: 3.43, 95% CI 1.95-6.05), and those engaged in the service sector such as family business or government employee (OR: 2.50, 95% CI 1.13-5.56) were more likely to report depressive symptoms in pregnancy than house wives. Moreover, women with household food insecurity are nearly five times as likely to be depressed during pregnancy as compared to women from food secure households (OR: 4.60, 95% CI 2.75-7.70). Presence of intimate partner violence during pregnancy was also associated with an increased likelihood that a woman was depressed during pregnancy, although the association was marginally significant (Table 3).

**Table 3:** Unadjusted and adjusted odds ratios of women’s experience of maternal depressive symptoms by pregnancy intention, social support and other characteristics, Southwestern Ethiopia, 2012

**Variables**	** Depressed mood, OR (95% CI)**	
	**Unadjusted OR**	**Adjusted**^**1 **^**OR**
	**(95% CI)**	**(95% CI)**
Pregnancy intention		
Wanted (reference)	1.00	1.00
Mistimed	1.33(0.84-2.10)	0.97(0.56-1.66)
Unwanted	2.76(1.61-4.70)***	1.96(1.04 -3.69)*
Social support		
Low (reference)	1.00	1.00
Medium	0.17(0.10-.31)***	0.27 (0.14-0.53)***
High	0.14(0.07-.25)***	0.23(0.11-0.47)***
Educational status		
No education (reference)	1.00	1.00
Primary	0.46(0.26-.79)*	0.56(0.30-1.05)
Secondary & above	1.10(0.46-2.66)	1.83(0.64-5.27)
Wealth tertile		
Lower (reference)	1.00	1.00
Middle	1.16 (0.73-1.85)	1.43 (0.82-2.51)
Upper	0.73(0.44-1.20)	0.88(0.48-1.62)
Occupation		
Housewife (reference)	1.00	1.00
Farmer	4.54(2.80-7.37)**	3.43(1.95-6.05)**
Employed/family business	2.09(1.06-4.14)*	2.50(1.13-5.56)*
Perceived work burden in pregnancy		
Too difficult (reference)	1.00	1.00
Moderate	0.56(0.34-.92)*	0.72(0.41-1.26)
Easy	0.49(0.30-.81)**	0.68(0.38-1.19)
Food insecurity	6.79(4.31-10.72)***	4.60(2.75-7.70)***
History of miscarriage or stillbirth	2.62 (1.47-4.67)**	1.27(0.62-2.57)
Partner violence during pregnancy	3.75(1.42-8.92)**	3.41(1.18-9.10)**

^1^Adjusted for education, wealth tertile, occupation, perceived work burden, food security status, history of miscarriage or still birth and partner physical violence during pregnancy.

*p < 0.05 **p < 0.01, ***p < 0.001.

## Discussion

The magnitude of antenatal depression in the current study population, 19.9% (95% CI, 16.8-23.1), though within the range of findings reported from Sub-Saharan Africa and other developing countries [11-17], is high when compared to findings from a systematic review that showed prevalence of prenatal depression of 12.0% in developed countries [10]. In Sub-Saharan Africa, the prevalence of antenatal depression ranged from 8.3% to 39% [16-18]. A very high level of depression was reported in a recent study in Cape Town, South Africa, where depressed mood in pregnancy was 39%. There has been no study of antenatal depression in Ethiopia, but in one study that used the Hopkins symptoms checklist (HSCL) to measure the prevalence of postnatal maternal and paternal symptoms of anxiety and depression, the prevalence of depression (defined as mean score for each HSCL item of ≥1.75) among adult women was 37% [20].

In this study, factors that were significantly associated with depressed mood in pregnancy include pregnancy intention, social support, occupation, food security status and partner violence during pregnancy. With regards to pregnancy intention, having unwanted pregnancy, not mistimed pregnancy, is associated with antenatal depression. Women reporting unwanted pregnancy are nearly 2 times more likely to be depressed as compared to women with planned pregnancies. Several previous studies have shown such an association between unwanted pregnancy and depression during pregnancy [32,34,35]. However, women reporting mistimed pregnancies did not differ significantly from women with wanted pregnancy in terms of depressive symptoms during pregnancy.

The strongest association in this study was with social support. In this study, women with high social support were 0.26 times as likely as women with low social support to experience antenatal depression. Similarly, women with moderate social support were 0.27 times as likely as women with low social support to experience antenatal depression. The association between social support and depression during pregnancy has been confirmed by studies from both developing and developed countries. These studies have shown that social support plays a buffering role from stressful life events by providing resources, support and strength during pregnancy [21,39]. Much related to the absence of social support is the presence of intimate partner violence during pregnancy. In this study, although very few women (about 4%) reported ever been beaten during the current pregnancy, there was a significant association between intimate partner violence and depression during pregnancy, as has been found in several previous studies [26,27].

Household level food insecurity is another important associated factor with depressed mood in pregnancy. About 41% of women in this study reported food insecurity, which can be one main cause of stress in life. Consequently, women reporting food insecurity are nearly five times as likely as food secure women to report depressive symptoms during pregnancy. Food insecurity is a major problem in Ethiopia and the study area in particular [20]. Moreover, this study took place in the summer months of June and July and in rural Ethiopia, these are times when most households run out of food, and food insecurity tends to be high during this season. Studies have also indicated that the effects of food insecurity extend beyond the nutritional effects and include anxiety and depression [20].

Our result indicates that socio-demographic factors such as age, parity, place of residence and wealth were not associated with prenatal depression. Similarly, the association between education and depressive symptoms was attenuated once the effects of other socio-demographic and obstetric factors were controlled for. Although such factors were found to be associated in some previous studies [17,25], a systematic review proved that such factors (age, parity, socio-economic status, and education) were not significant in multivariate models in majority of the studies included in the review [34].

### Limitations of the study

Despite the contributions that it makes to the literature on antenatal depression, this study has some limitations. First, although it examines the influences of unwanted pregnancy and social support on depressive symptoms during pregnancy, the study has not considered the presence of other important mental health conditions such as anxiety and stress in pregnancy. Second, given the nature of the study, a cross-sectional study, it is not possible to establish causal relationships. There is also a possibility of recall bias when reporting pregnancy intention. Moreover, standard instrument was not used to measure the variable ‘partner physical violence’ during pregnancy.

## Conclusion

Overall, our study found a high level of depressive symptoms among pregnant women in the study area. Although the study lacks clinical validation, the EPDS has been validated in several settings among pregnant women and is found to be a valid screening instrument for depressive symptoms during pregnancy. As shown above, much of these stressful life experiences may stem from the socio-economic context in which women live such as food insecurity, intimate partner violence and unwanted pregnancy. Understanding the factors that buffer the effects of stressful life events on depression in pregnancy is important. This study demonstrated that social support during pregnancy plays such a buffering role against depression. It is therefore important to screen for depression during pregnancy and provide appropriate counseling during routine prenatal care visits. The WHO has made such recommendations, integration of mental health into primary health care settings in developing countries [50]). In conclusion, enabling women to meet their reproductive goals and interventions that encourage social support in pregnancy help a lot in reducing mental health problems such as depression.

## Endnotes

^a^ Analysis of 2011 EDHS data for pregnant women showed that 32% of pregnancies were not intended. But, for births in the five years before the survey, 25% of them were reported as unintended.

^b^ Data from DSS registration for the year 2012.

^c^ Kebele is the smallest administrative unit in Ethiopia.

## Abbreviations

CSA: Central Statistical Authority; DSS: Demographic Surveillance Site; EPDS: Edinburgh Postnatal Depression Scale; OR: Odds Ratio; WHO: World Health Organization.

## Competing interests

We declare that we have no any competing interests.

## Authors’ contributions

YD designed the study, monitored the data collection, analyzed the data, and wrote the first draft of the manuscript. MF and MJH participated in the design of the study, supervised the whole process and reviewed and modified the drafts of the manuscript. All authors revised and approved the final version of this manuscript.

## Pre-publication history

The pre-publication history for this paper can be accessed here:

http://www.biomedcentral.com/1471-2393/13/135/prepub

## Supplementary Material

Additional file 1 Questionnaire for baseline survey among pregnant women in Gilgel Gibe HDSS area, SW Ethiopia. Click here for file
