# Supplementary material for: The association of unwanted pregnancy and social support with depressive symptoms in pregnancy: evidence from rural Southwestern Ethiopia
Source: BMC Pregnancy Childbirth. 2013 Jun 24;13:135. doi: 10.1186/1471-2393-13-135 (PMC3716614; doi:10.1186/1471-2393-13-135)
Supplement: Additional file 1 — Questionnaire for baseline survey among pregnant women in Gilgel Gibe HDSS area, SW Ethiopia. [file 1471-2393-13-135-S1.pdf]

Questionnaire for baseline survey among pregnant women in Gilgel Gibe HDSS area, SW Ethiopia.

Name of interviewer:	Kebele name:	Household ID:
Interviewer signature	House number:	Women ID:
Name of supervisor:		
Outcome of first visit	1. Completed 2. Not around home 3. Refused	Date of appointment for other time visit
Outcome of second visit	1. Completed 2. Not around home 3. Refused	Date of appointment for other time visit
Outcome of third Visit	1. Completed 2. Not around home 3. Refused	
Date of interview date/month/ year	-------- /--------/-----------	

	PART I: **SOCIO- DEMOGRAPHIC CHARACTERISTICS OF RESPONDENTS**		
101	In what month and year were you born?	Date ____________Month s ____________<br>Year ___________	
102	How old were you at your last birthday?	___________________	
103	Have you ever attended formal school?	Yes . . ……………………………….….. . . . 1<br>No . . . . ……. . .. …………………..… 2 →	105
104	What is the highest grade you completed?	Grade ______________________<br><br>technical / vocational certificate.........................1<br>university/ college diploma.............................. 2<br>university/college degree...................................3	
105	What is your religion?	Muslim……..………..……..……..………..……1<br>Protestant. ……………………..…………..……….2<br>Orthodox………………………..…………..……3<br>Catholic ……………………………………….4<br>Other __________________________ (Specify)	
106	What is your ethnicity?	Oromo…………………….........……...……1<br>Amhara …..………..……..……..……..…..……2<br>Yem ……………….……..…..……..…..…...….3<br>Dawro ……………………....………………..…4<br>Other_________________________ (Specify)	
107	What is your marital status?	Married--1<br>Widowed--2<br>Divorced--3<br>Separated---4	

108	What was the age at which you married for the first time?	______________________	
109	What is your usual occupation, that is, what kinds of work do you mainly do?<br>______________________	Housewife---1<br>farmer ---2<br>Government/NGO employee--------------------3<br>Merchant--4<br>Student --5<br>Other__________________________ (Specify)	
110	What is the main source of drinking water for members of your household?	piped into dwelling/compound……………..…1<br>piped outside compound …………..….………..2<br>open well ………………………….…..………..3<br>open spring ……………………………..……4<br>covered well …………………………………..5<br>covered spring ………………….…..………...6<br>surface water (river, pond or lake)……...........7<br>rain water ……………………………………..8<br>other __________________________ (Specify)	
111	What kinds of toilet facility do most members of your household use?	flush toilet ....1<br>traditional pit toilet...2<br>ventilated improved pit …………..…..….……3<br>latrine (vip)………………………….............4<br>no facility/bush/field ....5<br>other__________________________ (Specify)	
112	Does your household have?<br><br>Radio<br>Electricity<br>Television<br>Telephone/cell phone<br>Refrigerator<br>An electric mitad<br>A kerosine lamp/pressure lamp<br>Own the house living in?<br>Own a bicycle<br>A motorcycle<br>A car or truce<br>Farm land<br>Have cattle<br>How many cattle?<br>Have horse/ donkey<br>How many?<br>Have sheep/goats?<br>How many?	Yes No<br><br>1 2<br>1 2<br>1 2<br>1 2<br>1 2<br>1 2<br>1 2<br>1 2<br>1 2<br>1 2<br>1 2<br>1 2<br>1 2<br><br>1 2<br><br>1 2	
113	Main floor material of the house	earth/sand...1<br>dung..2<br>wood planks..3<br>reed/bamboo...4<br>parquet or polished wood..................................5<br>vinyl sheets/tiles...6<br>cement ....7<br>cement tiles/brick ....8<br>carpet..9<br>other __________________________	

114	How many rooms in your house are used for sleeping?	No. of rooms __________________	
115	Main roof material of your house	corrugated iron.. 1<br>Cement/concrete.. 2<br>wood and mud .... 3<br>thatch..4<br>reed/bamboo...5<br>plastic sheet..6<br>mobile roofs of nomads ....7<br>other __________________________________ 96	
116	How many people live in your household?	__________________________	

	**PART II: FERTILITY:** Now, I would like to ask you about the births that you have had in your life.		
201	How many children have you given birth to (male and female) in your life who are still alive?	Total ________________<br>Male _________________<br>Female ________________	
202	Have you ever given birth to a boy or girl who was born alive but later died?	Yes-------------------------------1<br>No ----------------------------- 2 →	204
203	How many such sons & daughters died?	Sons who died _____________<br>Daughters who died _________	
204	In total how many children have you given birth to in your lifetime?	_______________________	
205	What was your age when you gave birth to your first child?	___________________	
206	How many children did you give birth to in the last 5 years?	____________________	
207	Did you give birth to a child in the last 12 months?	Yes---------------------------------1<br>No ------------------------------- 2	
208	When was the last time you gave birth?	Month _____________________<br>Year _______________________	
209	**INTERVIEWER:** record the names of all live births (male and female) the woman had in the last five years whether still living or not, starting with the latest birth.		

	Name (start from the youngest)	Were any of these births twins?	Sex	Date of birth d/m/year	Age at his/her last birth day	Is (Name) alive or dead?	date of death
01		Single 1<br>multiple 2	male 1<br>female 2			Alive 1<br>Dead 2	
02		Single 1<br>multiple 2	male 1<br>female 2			Alive 1<br>Dead 2	
03		Single 1<br>multiple 2	male 1<br>female 2			Alive 1<br>Dead 2	
04		Single 1<br>multiple 2	male 1<br>female 2			Alive 1<br>Dead 2	

210	**INTERVIEWER:** Refer to Qn.209 and record the names of all live births (male and female) the woman had in the last five years whether still living or not, starting with the latest birth.	

	Enter names in Qn. 210.		
	Name of last child, next to last child, etc.	At the time you became pregnant with (Name), did you want to become pregnant then, did you want to wait until later, or did you not want to have any more children at all?	
01		wanted then ………………………….……..……..1<br>wanted later ……………………………………….…2<br>not wanted at all ………………………………..…….3	
02		wanted then ……………………………...……..1<br>wanted later ……………………………………….…2<br>not wanted at all ………………………………..…….3	
03		wanted then ……………………………..…..…..1<br>wanted later ……………………………………….…2<br>not wanted at all ………………………………..…….3	
04		wanted then ……………………………..…..…..1<br>wanted later ……………………………………….…2<br>not wanted at all ………………………………..…….3	

211	Have you ever had a pregnancy that was miscarried, was aborted, or ended in a stillbirth?	Yes ……………………..……..1<br>No ……….………….…………...2	→214
212	How many pregnancies did you have that did not end in a live birth?	No. of pregnancies________	
213	When did your last menstrual period come?	Days ago ________<br>Weeks ago _________<br>Months ago __________<br>Years ago ___________<br>Other ____________ (specify)	
214	If you could choose exactly the number of children to have in your whole life, how many would that be?	Number ________________<br>How many boys ___________<br>How many girls ____________	

	**PART III: Current Pregnancy:** Now I would like to ask you about the current Pregnancy		
301	Including this pregnancy, how many times have you been pregnant in your life?	No. ________________	
302	How many months/weeks pregnant are you now?	___________________	
303	How many weeks or months pregnant were you when you knew that you were pregnant? (For example, you had a pregnancy test or a doctor or nurse said you were pregnant.)	Weeks ________________<br><br>Months _______________	
304	At the time you became pregnant with this pregnancy; did you want to become pregnant then, Did you want to wait until later, or did you not want to have any more children??	wanted then ……………………..…..1<br>wanted later …………………………2<br>not wanted at all …………………….3	
305	In the month that you became pregnant.....	You were not using contraception ……..………...1<br>You were using contraception, but not on every occasion………………………..……………….2<br>You always used contraception, but knew that the method had failed ....3<br>You always used contraception ………...........4	

306	How happy are you to be pregnant?	Very unhappy……………………………………………1<br>Unhappy………………………………………………2<br>Neither happy nor sad……………………………3<br>Happy…………………………………………………4<br>Very happy…………………………………………..5	
307	Thinking back to just before you got pregnant, how did you feel about becoming pregnant?	I wanted to be pregnant sooner-……………………1<br>I wanted to be pregnant later-………………………2<br>I wanted to be pregnant then ………………………3<br>I didn't want to be pregnant then or at any time in the future………………………………………………4	
308	When you got pregnant with your new baby, Were you or your husband or partner doing anything to keep from getting pregnant? (ex. using birth control methods.)	Yes ………………………………………………1<br>No ………………………………………………2	
309	Which of the following methods were you/your husband using before you got pregnant?	Pills …………………………………………………1<br>Injectbales…………………………………………2<br>IUD……………………………………………………3<br>Norplants ……………………………………………4<br>Condoms ……………………………………………5<br>LAM……………………………………………………6<br>Sterilization-…………………………………………7<br>Foam/jelly-……………………………………………8<br>SDM…………………………………………………… 9<br>Rythum method…………………………………10<br>Other ___________________________ (specify)	
310	Now I have some questions about the future. After the child you are expecting now, would you like to have another child, or would you prefer not to have any more children?	have a/another children…………………………1<br>No more ……………………………………………2<br>Undecided/don't know……………………………3<br>can't get pregnant -…………………………………4<br>Unsure ………………………………………………5	
311	After the child you are expecting now, how long would you like to wait before the birth of another child?	Months __________________<br>Years ___________________<br>Soon/now ……………………………………………1<br>Don't know …………………………………………2	

Part IV : Social Support and Feelings of Depression .

Now, we would like to ask you questions about the support you get from different people and the way you have been feeling in the past weeks

400.	For each of the following statements, please give your opinion as to which shows how you feel about the support you have now.	Always	Most of the time	some of the time	rarely	Never

1	I have good friends who support me	5	4	3	2	1
2	My family is always there for me	5	4	3	2	1
3	My husband/partner helps me a lot	5	4	3	2	1
4	There is conflict with my husband/partner	1	2	3	4	5
5	I feel controlled by my husband/partner	1	2	3	4	5
6	I feel loved by my husband/partner	1	2	3	4	5

Q401-410. Feelings of depression. Tell us the way you have been feeling in the past (1) week including today. In the past seven days,

401	You have been able to laugh and see the funny side of things	As much as I always could……………………….….………..0<br>Not quite so much now……………………….….…………1<br>Definitely not so much now ……………….…………………2<br>Not (hardly) at all …………………………….……….....3	
402	You have looked forward with enjoyment to things	As much as I ever did …………………….….…........0<br>Rather less than I used to ………….……………….....1<br>Definitely less than I used to ………..…….….……...2<br>Hardly at all……………………..………..…….….…3	
403	I have blamed myself unnecessarily when things **went wrong**	Yes, most of the time………………..……..….….……0<br>Yes, some of the time ……………………...….…………1<br>Not very often ……………………………….…… 2<br>No, never ………………………..…….….……...……..3	
404	I have been anxious or worried for no good reason	No, not at all ……………………………….……..…0<br>Hardly ever ………………………….…..…………1<br>Yes, sometimes …………………..….….…………2<br>Yes, very often……………………......…………3	
405	I have felt scared or panicky for no very good **reason**	No, not at all ………………………………..…...…0<br>No, not much …………………………...…...….…1<br>Yes, sometimes ……………………………….….……2<br>Yes, quite a lot ……………………………..…...……3	
406	Things have been getting on top of me	No, I have been coping as well as ever ……………..……..0<br>No, most of the time I have coped quite well …….……....1<br>Yes, sometimes I haven't been coping as well as usual..….2<br>Yes, most of the time I haven't been able to cope at all ..…3	
407	I have been so unhappy that I have had difficulty **sleeping**	No, not at all ……………….………...…….….……..…0<br>Not very often ……………………...……….….….…1<br>Yes, sometimes ……………………..……..…....…2<br>Yes, most of the time ………………..………..…….…3	
408	I have felt sad or miserable	No, not at all …………………..………..……….…..…0<br>Not very often ………………..…..……………..…….1<br>Yes, sometimes ……………………..……..……….…2<br>Yes, most of the time ……….……………….………3	

409	I have been so unhappy that I have been crying	No, never ……………………….….……..……....…0<br>Only occasionally ………………..…..………...…..…..…1<br>Yes, quite often …………………..………..……..…..…2<br>Yes, most of the time ……………………….…....…..…3	
410	The thought of harming myself has occurred to me	Never ……………………….……….....……..……….0<br>Hardly ever ……………………….…..……..……....…1<br>Sometimes ………………………….……..……..…....…2<br>Yes, quite often ………………………..…..…...…....…3	
411	In general, how do you describe your overall health now?	Excellent …………………………………….…………….1<br>Very good ………………………………..…………..2<br>Good ……………………………..………..……….3<br>Fair ……………………….…………….……..………..4<br>Good ……………………………………………….5	

NO	**PART V: Maternal Health Care Utilization**	
501	After you knew that you are pregnant, did you go any where to receive antenatal care?	Yes 1<br>No 2 → 507
502	How many weeks or months pregnant were you when you first received antenatal care for the pregnancy?	Number of months…………….. |___|___| Weeks....................................
503	How many times did you receive antenatal care during the current pregnancy?	Number …………….. |___|___| Don't know...................................11
504	Where did you receive antenatal care for the current pregnancy?	Hospital ……………………….……....…..….…1<br>Health center ………………….…..…...…..... 2<br>Health post ………………….……….……..… 3<br>Other public facility ……………..…..….....…4<br>Private hospital/clinic …………..……......…....5<br>NGO health facility ………………...……….…..6<br>Home ………………………….……..……..........7
505	Whom did you see for antenatal care?	Doctor …………………………………………1<br>Nurse/Midwife…………………………………2<br>Other health professional ……….……..……3<br>TTBA ……………………….…………..…..…4<br>Untrained TBA …………………..…..……...5<br>Community health agent…….………….……...6<br>Other _________________________(specify)
506	During this pregnancy, were you given an injection in the arm to prevent the baby from getting tetanus, that is, convulsions after birth?	Yes --------------------------------1<br>No ----------------------------------2
507	During this pregnancy, how many times did you receive tetanus injection?	____________________
508	During any of your prenatal care visits did a doctor, nurse, or other health care worker talk with you about any of the things listed below?<br>a. How drinking alcohol or smoking cigarettes during pregnancy could affect your baby<br>b. The signs and symptoms of preterm labor (labor more	Yes No<br>1 2<br>1 2

	than 3 weeks before the baby is due)<br>c. Signs of pregnancy Complications?<br>d. What to do if your labor starts early<br>e. What to do for Pregnancy complication<br>f. Getting tested for HIV (the virus that causes AIDS)<br>g. Where to go for delivery<br>h. Breastfeeding your baby after birth	1 2<br>1 2<br>1 2<br>1 2<br>1 2<br>1 2<br>1 2<br>1 2
509	Can you name any danger signs during pregnancy, childbirth, or soon after delivery that indicate a woman has a serious health problem?<br>________________________________<br><br>IF YES: Which ones?	excessive bleeding...1<br>baby in bad position...2<br>Swelling of the hands or face……………..….. 3<br>Convulsions/fits, eclampsia............................... 4<br>fever...5<br>prolonged labor ....6<br>placenta retained ....7<br>foul vaginal discharge .... 8<br>anemia .... 9<br>Leaking urine ....10<br>leaking stools ....11<br>other ___________________________<br>(specify)<br>don't know..
510	Do you think a woman could die from any of these causes?	Yes ---1<br>No --2
511	What is the closest health facility at which a woman can go to deliver a baby with assistance from a doctor, nurse, or midwife?<br><br>What is the name of the facility? ________________	Gov't hospital…………......…......…1<br>Gov't health center ……………..…2<br>Health post ……………...…..…...3<br>Private hospital……………..…..…4<br>Private clinic ……………..…..…5<br>other ________________ (specify)
512	How far is it from here to facility (mentioned in q.414)?	Distance in kms_________<br><br>Do not know........................
513	How long does it take to **walk** from here to facility (mentioned in q.414)?	Minutes................................<br>Don't know minutes...................
514	Where do you want to give birth to your baby for the current pregnancy?	Home ……………..…..……………1<br>Health post …………..…..……………2<br>Health center ……..……..…..……..3<br>Hospital……………….…….…..…4<br>Private clinic ……….……..……....5
515	If your answer to q 417 is 5 (home), why didn't you deliver in a health facility?	Costs too much………….……….....1<br>Facilities not open …..………..…….2<br>Have no health problem …………… 3<br>Too far from home ……………........4<br>No transportation……......………….5<br>Don't trust facility ……….............…6<br>No female provider ………………..7<br>Husband/family did not allow ....8<br>Not customary …………...….........9<br>Other __________________ (specify)
516	Who would you prefer to assist you during your delivery?	Doctor ………………...…..……..1<br>Nurse/midwife ………………….…2<br>Health extension worker ………..…3<br>Other health professional …………4<br>TTBA ……………………….…...5

		Relative…….………………..……….6<br>Other ________________ (specify)
517	Did you ever have a baby by cesarean delivery or c-section (When a doctor cuts through the mother's belly to bring out the baby)?	Yes ……………………..…………1<br>No ………….………..................…2
518	How was your ***last*** baby delivered?	Has no previous birth ………..……..1<br>Vaginally……………………..……..2<br>Cesarean delivery (c-section)……... 3
519	Did you have any of the following problems during the current pregnancy?<br>Vaginal bleeding<br>Severe headache<br>Severe nausea, vomiting, or dehydration<br>High blood pressure, hypertension<br>Other ______________________	Yes No<br><br>1 2<br>1 2<br>1 2<br>1 2
520	Did you seek treatment for any of these problems	Yes ……………………..…………1<br>No ………….………..................…2
521	During the last 3 months of your recent pregnancy,<br>Did you smoke cigarettes, Gaya or Shisha in an average week?<br>Did you drink alcoholic drinks in an average week?<br>Did you chew chat in an average week?	Yes No<br>Cigarette 1 2<br>Alcohols 1 2<br>Chat 1 2
522	At any time during your **current pregnancy**, did your husband push, hit, slap, kick, choke, or physically hurt you in any other way?	Yes ……………….……….................1<br>No …………………….…….……...2

PART VI: Husband's Characteristics and Women's Autonomy			
600	Are you married or currently living with a partner?	Yes ………………….……..………..1<br>No ………………….……..……….. 2	→604
601	How old was your husband/partner on his last birthday?	Age ________________	
602	What is your husbands educational status	None ………………………….……… ….0<br>Grade ____________________<br>technical / vocational certificate................1<br>university/college diploma........................2<br>university/college degree...........................3	
603	What is your husbands occupation	Farmer -………………….……..………..1<br>Civil servant……………….…………….2<br>Merchant-……………….……..……….. 3<br>Student……………….……..………..4<br>Other ______________________ (specify)	
604	Some women take up jobs for which they are paid in cash or kind. Others sell things, have a small business or work on the family farm or in the family business.<br>Are you currently doing any of these things or any other paid work?	Yes ………………….……..………..1<br>No ………………….……..……….. 2	
605	What is your occupation, that is, what kind of work do you mainly do?	____________________________	

606	Are you paid in cash or kind for this work or are you not paid at all	Cash only -----------------------------------1<br>Cash and kind ------------------------------2<br>In kind only --------------------------------3<br>Not paid ------------------------------------4	
607	Who decides how the money you earn will be used: mainly you, mainly your husband/partner, or you and your husband/partner jointly?	You ---1<br>Husband/partner --------------------------2<br>You & your husband jointly -------------3<br>Other (specify)-------------------- --------4	
608	Who decides how the money your husband earns will be be used: mainly you, mainly your husband/partner, or you and your husband/partner jointly?	You ---1<br>Husband/partner ---------------------------2<br>You & your husband jointly -------------3<br>Other (specify)---------------- ------------4	
609	Who usually makes decision about health care for Yourself :mainly you, mainly your husband, you and your husband jointly, or someone else	You ---1<br>Husband/partner ---------------------------2<br>You & your husband jointly -------------3<br>Other (specify)------------------ ---------4	
610	Who usually makes decision about making major Household purchases?	You ---1<br>Husband/partner ---------------------------2<br>You & your husband jointly -------------3<br>Other (specify)----------------------- ----4	
611	Who usually makes decision about making purchases for Daily household needs?	You --1<br>Husband/partner --------------------------2<br>You & your husband jointly ------------3<br>Other (specify)-------------------- ------4	
612	Who usually makes decision about visits to your family or relatives	You --1<br>Husband/partner --------------------------2<br>You & your husband jointly ------------3<br>Other (specify)----------------- ----------4	

**PART VII:** Household food security and work burden			
701	In the last three months, did you ever worry that your household would not have enough food?	Yes …………….……….………..1<br>No ………………………………… 2	
702	In the last three months, did you ever reduced of balanced diet to your child because of lack of food or money to buy	Yes …………….……….………..1<br>No ………………………………… 2	
703	In the last three months, did you ever reduce the number of meals for in your child a day because of shortage of food or money	Yes …………….…..……………..1<br>No ……………………….………… 2	
704	In the last three months, did you ever reduce the number of meals of eaten child a day because of shortage of food or money	Yes …………….…..……………..1<br>No ……………………….………… 2	
705	In the last three months, did you ever spend the whole day without eating because of shortages of food or money	Yes …………….…..……………..1<br>No ……………………….………… 2	

706	In the last three months, did you ever ask for food or money to buy food?	Yes …………………..…..……………..1<br>No ………………………….……………… 2	
606	What type of works, including household chores such as fetching water, cooking food…. etc, did you do in the last 24 hours?<br><br>1. Did you cook food for the family?<br>2. Did you fetch water from a stream or river?<br>3. Did you collect fuel wood from the bush?<br>4. Did you carry heavy materials<br>5. Did you travel long distance (such as to market place)<br>6. Did you work on the farm?<br>7. Any other ______________________	Yes No<br><br>1 2<br>1 2<br>1 2<br>1 2<br>1 2<br><br>1 2	
607	How do you describe the works you did in the last 24 Hours?	Were difficult…………………..…..…………1<br>Neither difficult nor easy……….….……2<br>Were easy …………………...…………..3	

SECTION VIII: Maternal Anthropometry

**H1**	**Weight**	**------------ Kg**
**H2**	**Height**	**-------------- cm**
**H3**	**MUAC**	**--------------- cm**
